# Phytoplankton-Specific Response to Enrichment of Phosphorus-Rich Surface Waters with Ammonium, Nitrate, and Urea

**DOI:** 10.1371/journal.pone.0053277

**Published:** 2013-01-17

**Authors:** Derek B. Donald, Matthew J. Bogard, Kerri Finlay, Lynda Bunting, Peter R. Leavitt

**Affiliations:** 1 Limnology Laboratory, Department of Biology, University of Regina, Regina, Saskatchewan, Canada; 2 Department of Biology, University of Regina, Regina, Saskatchewan, Canada; Consiglio Nazionale delle Ricerche (CNR), Italy

## Abstract

Supply of anthropogenic nitrogen (N) to the biosphere has tripled since 1960; however, little is known of how *in situ* response to N fertilisation differs among phytoplankton, whether species response varies with the chemical form of N, or how interpretation of N effects is influenced by the method of analysis (microscopy, pigment biomarkers). To address these issues, we conducted two 21-day *in situ* mesocosm (3140 L) experiments to quantify the species- and genus-specific responses of phytoplankton to fertilisation of P-rich lake waters with ammonium (NH_4_^+^), nitrate (NO_3_^−^), and urea ([NH_2_]_2_CO). Phytoplankton abundance was estimated using both microscopic enumeration of cell densities and high performance liquid chromatographic (HPLC) analysis of algal pigments. We found that total algal biomass increased 200% and 350% following fertilisation with NO_3_^−^ and chemically-reduced N (NH_4_^+^, urea), respectively, although 144 individual taxa exhibited distinctive responses to N, including compound-specific stimulation (*Planktothrix agardhii* and NH_4_^+^), increased biomass with chemically-reduced N alone (*Scenedesmus* spp., *Coelastrum astroideum*) and no response (*Aphanizomenon flos-aquae*, *Ceratium hirundinella*). Principle components analyses (PCA) captured 53.2–69.9% of variation in experimental assemblages irrespective of the degree of taxonomic resolution of analysis. PCA of species-level data revealed that congeneric taxa exhibited common responses to fertilisation regimes (e.g., *Microcystis aeruginosa*, *M*. *flos-aquae*, *M*. *botrys*), whereas genera within the same division had widely divergent responses to added N (e.g., *Anabaena*, *Planktothrix*, *Microcystis*). Least-squares regression analysis demonstrated that changes in phytoplankton biomass determined by microscopy were correlated significantly (*p<*0.005) with variations in HPLC-derived concentrations of biomarker pigments (*r* ^2^ = 0.13–0.64) from all major algal groups, although HPLC tended to underestimate the relative abundance of cyanobacteria. Together, these findings show that while fertilisation of P-rich lakes with N can increase algal biomass, there is substantial variation in responses of genera and divisions to specific chemical forms of added N.

## Introduction

Human activities such as farming and industrial fixation of atmospheric nitrogen (N) have tripled the supply of N to the biosphere since 1960 and are expected to double present levels of N influx by 2050 to meet future demands for food production [1], [2]. In particular, application of N fertilisers will exceed 275 Tg N year^−1^ and will be concentrated in regions where centuries of farming may have saturated soils with phosphorus (P) [3], [4], increased P export to lakes [5], and overloaded surface waters with P [6]. In these regions, lakes already exhibit poor predictive relationships between P influx and algal abundance [7], continuously high concentrations of dissolved P despite abundant phytoplankton [3], [8], insufficient biological fixation of N to support primary production [9], [10], and strong positive correlations between N influx and total algal or cyanobacterial abundance [6], [11]. Taken together, these findings suggest that persistent fertiliser application has weakened the regulatory role of P [12], [13], and that pollution with N may further degrade water quality in P-rich lakes [11].

At present, most evidence shows that freshwater eutrophication ultimately arises from persistent increases in P influx from urban and other anthropogenic sources [14]–[16]. However, synthesis of laboratory studies [17], [18], *in situ* mesocosm experiments [19], [20], whole-ecosystem manipulations [21] (but see) [22], catchment-scale mass balances [11], [23], regional surveys [24] and palaeolimnological reconstructions [6], [11] also demonstrates that rises in N influx can independently increase algal biomass, alter the proportion of diazotrophic cyanobacteria, and increase toxicity of some algae, particularly in lakes with total P (TP) concentrations over 100 *µ*g P L^−1^ and N : P mass ratios below ∼20∶ 1 [25]. Lack of reconciliation between these two robust data sets has resulted in vigorous and occasionally acrimonious debate over the unique and interactive roles of N in regulating baseline lake productivity [26], [27] and cultural eutrophication [13], [28], [29].

Continuing uncertainty over ecosystem consequences of N pollution may arise in part because of comparatively limited understanding of how effects of N may vary with phytoplankton identity, nutritional capability and status, and the chemical form of added N [18], [30], [31]. At a coarse taxonomic level, preliminary evidence suggests that fertilisation of eutrophic waters with ammonium (NH_4_^+^) or urea ([NH_2_]_2_CO) increases the *in situ* abundance of non-N-fixing cyanobacteria [20], [25] which exhibit efficient light use [32] and superior NH_4_^+^ uptake kinetics [33], as well as chlorophytes which can sustain rapid growth using diverse N sources if sufficient light is present [34], [35]. In contrast, the competitive advantage of diazotrophic cyanobacteria can be lost following N fertilisation because N uptake suppresses formation of heterocysts and nitrogenase enzyme complexes needed for biological fixation of N_2_ [20], [36]. Finally, addition of nitrate (NO_3_^−^) to P-rich waters can favour production of diatoms if silica (Si) is available [37], [38], possibly due to non-saturating uptake kinetics for this compound [39]. However, despite these broad generalizations, substantial uncertainty surrounds the *in situ* response of individual species or genera of phytoplankton to fertilisation with N [40]–[42] due to low taxonomic resolution of prior N studies (division-level), substantial overlap among algae in *in vitro* nutrient-uptake capabilities (N half-saturation constant, K_s_, = 1–14 *µ*g N L^−1^) and maximum growth rates (N-sufficient V_max_ = 0.2–8.0 ln units day^−1^) [30], [31], [43], and the high degree of environmental simplification in laboratory and microcosm studies [44].

In this study, we conducted 21-day long mesocosm (3140 L) experiments in summer and autumn to quantify the *in situ* response of over 140 individual phytoplankton taxa to enrichment of P-rich freshwaters with NH_4_^+^, NO_3_^−^ and urea. Effects of N addition on total algal abundance and gross community composition in this experiment have been analysed previously using high performance liquid chromatography (HPLC) and reported in [25]. Instead the unique objectives of the present study are four-fold: 1) to use microscopic analysis to quantify interspecific variation among algae in the response to N amendments; 2) to determine how phytoplankton-specific responses vary with the chemical form of added N; 3) to evaluate the influence of the taxonomic resolution of microscopic analysis (division, genus, species) on interpretation of N effects on phytoplankton, and; 4) to compare changes in phytoplankton assemblages derived from microscopic enumeration of cell densities and chromatographic analysis of algal pigments. As suggested elsewhere, biomarker-based analyses might be biased by limited taxonomic resolution, phylogenetic variation in cellular pigment content, or changes in ambient environmental conditions (light, temperature, nutrient availability) which uniquely influence the cellular quota of pigments [45]–[47]. Improved understanding of the nature of taxon-specific responses to N influx may help protect aquatic ecosystems against future pollution with agricultural N [2], optimise wastewater treatment procedures [28], and resolve on-going debate concerning the respective roles of N and P in regulating cultural eutrophication [48].

## Methods

### Study Site

**Figure 1:**
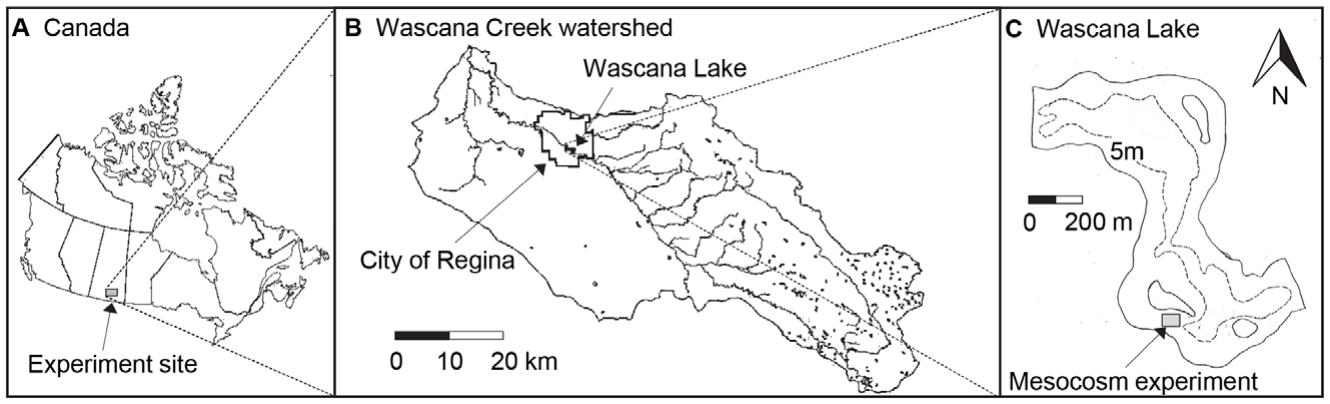
Map of Wascana Lake, Saskatchewan, Canada. Map shows a) the continental location, b) the gross drainage area (1400 km^2^) and c) depth contour map with the location of the mesocosm experiment (shaded box).

The experiments were conducted in Wascana Lake (Fig. 1), an unstratified, 0.5 km^2^, 7-m deep basin located within the central urban park of the City of Regina, Saskatchewan, Canada (50°26.17′N, 104°36.91′W). Regional evaporation (∼60 cm year^−1^) exceeds precipitation by two-fold, the climate is classified as sub-humid continental, and mean monthly air temperatures vary by up to 35°C (19°C in July, −16°C in January) [49]. Wascana Lake is fed by Wascana Creek, a permanent stream which drains a 1400 km^2^ agricultural basin [49]. Snow melt during March–April typically accounts for 80% of annual runoff in the region [50] resulting in seasonally variable, but generally low, water residence in the lake (decadal mean ∼0.07 yr) [49].

Wascana Lake has been monitored biweekly (May–Aug) since 1996 and exhibits elevated but annually-variable mean (±SD) summer concentrations of Chl *a* (39±48 *µ*g L^−1^), soluble reactive P (SRP) (192±161 *µ*g P L^−1^), total dissolved P (TDP) (299±208 *µ*g P L^−1^), NO_3_^−^ (119±217 *µ*g N L^−1^), NH_4_^+^ (94±203 *µ*g N L^−1^), total dissolved N (TDN) (1327±726 *µ*g N L^−1^), and dissolved organic carbon (DOC) (16.2±4.0 mg C L^−1^). Consequently, mean mass ratios of TDN : SRP are low (6.9±6.6). Typical plankton phenology includes a progression from a spring community composed of diatoms, cryptophytes, and copepods (*Diacyclops thomasi*, *Leptodiaptomus siciloides*), through a pronounced June clearwater phase with high densities of large-bodied Cladocera (*D. magna, D. pulicaria, D. galeata mendotae*) [51], to a summer and autumn community composed of small-bodied zooplankton (*Eubosmina coregoni, Bosmina longirostris, Diaphanosoma birgei, Daphnia retrocurva*, *Ceriodaphnia, Chydorus,* rotifers) and abundant cyanobacteria [8], [49]. Overall, cryptophytes, chrysophytes and chlorophytes comprise <20% of summer algal biomass. Instead, N-fixing *Aphanizomenon flos-aquae* and *Anabaena* spp. are abundant immediately after the clearwater phase, *Microcystis* spp. are common in the warm (>25°C) surface waters during August, *Planktothrix agardhii* is dominant during late-August through September, and *Phormidium* (and *Cyclotella*) spp. increase thereafter. Consequently, late-summer concentrations of the cyanobacterial toxin microcystin (MC) can be 10-fold greater than the upper limits recommended by the World Health Organization for drinking water (1 µg MC L^−1^).

### Mesocosm Experiments

Three-week long mesocosm experiments were conducted during both August and September of 2008, as described in [25]. Briefly, 2-m wide by 1-m deep, cylindrical, opaque white poly-weave enclosures (3140 L) were open to the atmosphere, closed to the sediments, and located in a sheltered embayment (Fig. 1). Enclosures were filled passively (drawn up from depth), attached to anchored floating frames, and assigned treatments at random. No attempt was made to circulate these well-mixed mesocosms or to modify zooplankton densities, although minnow traps were added to each enclosure and checked routinely to remove planktivorous fish. Advantages and limitations of this experimental design for eutrophication studies are detailed in [20] who studied division-level effects of urea on algal communities and [25] who also contrasted urea with nitrate and ammonium effects, but did not analyse species- and genus-level responses.

Each experimental treatment consisted of three replicates to which N was added on days 0, 7, and 14 as sodium nitrate (NaNO_3_), ammonium chloride (NH_4_Cl) or urea ((NH_2_)_2_CO). All trials received 6 mg N L^−1^ per week, whereas unamended enclosures served as controls. Nitrogen additions were intended to increase soluble N : P to >22∶ 1 by mass to suppress N-fixing cyanobacteria [52], were based on decadal mean concentrations of SRP and TDN, and were within the range of N content observed in regional lakes [50].

Sampling took place on days 0, 3, 7, 14 and 21 between 10 00 h and 14 00 h, immediately after N addition on day 0, and before N additions on days 7 and 14. Water was collected from mesocosms using a 6-L Van Dorn bottle deployed centrally at 0.5-m depth. All water samples were screened (247-µm mesh) in the field to remove invertebrates, filtered through 0.45-µm pore membrane filters within 2 hr, and stored in darkness at 4°C until analysed for chemical solutes. Samples of 100 mL were collected from Van Dorn bottles and preserved with Lugol’s iodine solution for microscopic analysis of phytoplankton species composition and abundance. In addition, particulate organic matter (POM) was concentrated on GF/C glass fibre filters (nominal pore 1.2-µm), and stored frozen (−10°C) in darkness until analysis of biomarker pigments using HPLC. Physical (Secchi disk transparency, temperature) and chemical parameters (conductivity, dissolved O_2_, pH) were also recorded in the field following standard protocols [11], [49] and are reported in [25].

### Laboratory Analysis

Preserved phytoplankton were identified, enumerated and measured using a Leica model DM IRB inverted light microscope (Leica Microsystems, Concord, Canada) and the Utermöhl sedimentation technique [53]. Aliquots of 2-mL were settled for 24 h prior to enumeration and analysis. Counts of cells, filaments and colonies were conducted at 100×, 400× or 1000× magnification, depending on the size and abundance of algal units, and were identified following the taxonomic conventions of [54]–[56]. Enumeration at 100× was made on every second transect within 50% of the depositional area of slides, those at 400× on one horizontal and one vertical transect, and counts at 1000× on 30 random fields of view, such that over 300 algal units were recorded for each sample. Cell measurements were conducted for abundant taxa at 1000× magnification on 30 randomly-selected individuals from a composite sample representing all enclosures and sampling events. Biovolume calculations followed [57] and were converted to cellular biomass by assuming that 1 mm^3^ of volume was equivalent to 1 mg of wet-weight biomass.

Phytoplankton pigments were extracted from POM using a mixture of acetone : methanol (80∶ 15, by volume) and were quantified using standard methods including spectrophotometry [58] and HPLC analyses [59]. HPLC used an Agilent model 1100 system (Agilent Technologies Inc, Mississagua, Canada) fitted with photodiode-array and fluorescence detectors and was calibrated using authentic pigment standards. Chlorophyll *a* (Chl *a*) concentrations were used to estimate total algal abundance (*µ*g L^−1^), while other taxonomically-diagnostic pigments (nmol L^−1^) were used to quantify changes in siliceous algae (mainly diatoms and chrysophytes) (fucoxanthin), cryptophytes (alloxanthin), dinoflagellates (peridinin), chlorophytes (Chl *b*) and colonial cyanobacteria (myxoxanthophyll). Pigments from N-fixing cyanobacteria (canthaxanthin, aphanizophyll) were near detection limits during the 2008 experiments and were not included in further analyses.

Concentrations of dissolved nutrients were conducted at the University of Alberta Water Chemistry Laboratory following [60], are reported in [25], and included TDP, SRP, TDN, NH_4_^+^+NH_3_ (as NH_4_^+^ hereafter), and NO_3_^−^+NO_2_^−^ (as NO_3_^−^ hereafter). Concentrations of urea [61] and dissolved organic carbon (DOC) [62] were determined at University of Regina Environmental Quality Analysis Laboratory using standard protocols reported in [25].

### Numerical Analysis

Repeated measures analysis of variance (RM-ANOVA) was used to estimate the statistical significance of differences in phytoplankton abundance among treatments, as well as interactions between time and treatment effects [20], [25]. Briefly, data were transformed (log_10_ [x+1]) as necessary prior to analysis and appropriate critical *F-*statistics selected for experiments with four treatment levels, three replicates and five sampling events (i.e. *F*_treatment_ = 4.07, *F*_time_ = 2.78, *F*_treatment×time_ = 2.18). Statistically-significant differences among treatments were tested using Tukey’s Honestly Significant Difference (HSD) *post hoc* test. No correction was made for the number of comparisons as one of our intentions was to quantify the general patterns of phytoplankton response to N amendments, although we recognize that this approach may inflate the number of apparently-significant algal responses. Least-squares regression analysis was used to quantify the linear relationship between microscopic- and pigment-based estimates of phytoplankton abundance. RM-ANOVA and linear regressions were conducted using software from SPSS version 11 (IBM, Armonk, NY, USA) and SYSTAT version 10 (SYSTAT Software Inc., Chicago, IL, USA), respectively.

Principal component analysis (PCA) was used to summarise the main patterns of phytoplankton community response to N fertilisation, and to evaluate how patterns of response varied with the taxonomic resolution of microscopic analysis, including division or class (division hereafter), genus and species. These levels of classification were selected because they are commonly used in limnological studies, but differ considerably in the total effort required for microscopic analysis [56]. An individual genus or species was included in PCA only if their mean biomass for the experiment was >1% of the total algal abundance in at least one of the twelve experimental enclosures. Estimates of algal abundance were log_10_ (x+1)-transformed prior to analysis using CANOCO version 4.5 software (Microcomputer Power, Ithaca, NY, USA). As our intention was to evaluate algal response to N fertilisation, categorical N treatments (e.g.,+or − urea) were included as passive variables in each PCA, whereas other environmental variables were not included in the ordinations.

This study obtained all necessary permits and approvals required by Environment Canada, Saskatchewan Environment, Transport Canada, and the Wascana Centre Authority (City of Regina) and adhered to all ethical and environmental regulations of the University of Regina and the Natural Sciences and Engineering Research Council of Canada.

## Results

### Lake and Mesocosm Conditions

As presented in [25], nutrient concentrations in Wascana Lake were elevated (∼125–175 µg P L-1, ∼1.2–1.6 mg N L-1), TDN : TDP mass ratios were low (∼10–15), and bottle bioassays revealed that phytoplankton growth exhibited instantaneous limitation by N supply during both August and September. As expected, mesocosm fertilisation elevated TDN to 15–20 mg N L-1, but had few measured effects on mesocosm water chemistry (pH, conductivity, oxygen, etc.) other than a rapid decline in SRP from ∼100 µg P L-1 to <5 µg P L-1, and a 50% decline in TDP, by day 14 in all N-amended enclosures. Further details and interpretations of water chemistry change are presented in [25].

### Community Response to N fertilisation

**Figure 2:**
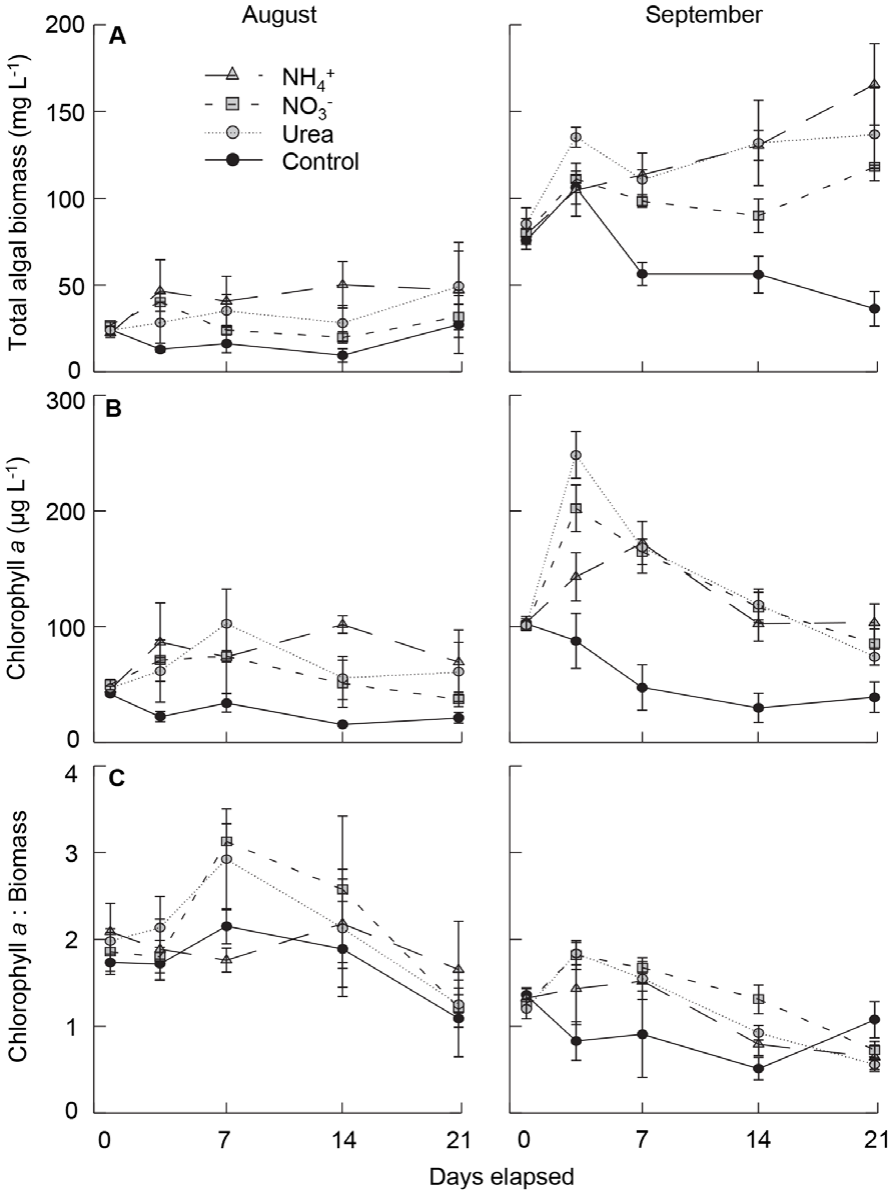
Biomass responses of total algal response to fertilisation with nitrogen in mesocosms conducted in August and September. Time series include; a) total phytoplankton biomass (mg wet mass L^−1^), b) Chl *a* (*µ*g L^−1^) and c) the ratio of Chl *a* : total phytoplankton biomass. Symbols represent mean and standard error (± SE, *n* = 3) for each nitrogen treatment, including amendments with NH_4_^+^ (shaded triangle, coarse dashed line), NO_3_^−^ (shaded square, medium dashed line) and urea (shaded circle, fine dashed line), as well as unamended (control) mesocosms (solid circle, solid line).

Total algal biomass measured microscopically or using ubiquitous Chl *a* increased ∼200% and ∼350% when fertilised with NO_3_^−^ and chemically-reduced N (urea, NH_4_^+^), respectively (Fig. 2). Biomass response to NH_4_^+^ fertilisation during August and all N amendments in September was statistically significant (*p*_treatment_ <0.05) relative to control enclosures (Table 1), although there was no significant effect of treatment on the ratio of Chl *a* : biomass (*p*_treatment_ >0.05).

**Table 1:** Repeated-measures analysis of variance of total phytoplankton biomass, chlorophyll *a*, chlorophyll *a* : biomass ratio, and biomass of major algal groups.

Response variable	August		September	
	*p*	*Post hoc*	*p*	*Post hoc*
Total phytoplankton biomass				
Treatment	**0.041**	NH, U, NO>U, NO, C	**0.000**	U, NH, NO>C
Interaction	**0.042**		**0.000**	
Chlorophyll *a*				
Treatment	**0.043**	NH, U, NO>U, NO, C	**0.000**	U, NO, NH>C
Interaction	**0.016**		**0.000**	
Chlorophyll *a* : biomass ratio				
Treatment	0.335	–	0.077	–
Interaction	0.457		**0.000**	
Cyanobacteria				
Treatment	0.110	–	**0.000**	NH, U, NO>C
Interaction	**0.035**		**0.000**	
Chlorophytes				
Treatment	**0.048**	NH, U, NO>U, NO, C	**0.001**	NH>U, NO, C
Interaction	0.053		**0.000**	
Diatoms				
Treatment	**0.004**	NO, U, C>C, NH	**0.009**	NO, U, C>U, C, NH
Interaction	**0.000**		**0.000**	
Chrysophytes				
Treatment	0.092	–	**0.005**	NH, U>U, C, NO
Interaction	**0.032**		**0.004**	
Cryptophytes				
Treatment	**0.025**	NO, U, C>U, C, NH	**0.020**	U, NO, C>NO, C, NH
Interaction	**0.007**		**0.002**	
Dinoflagelates				
Treatment	**0.009**	C, U, NO>NO, NH	0.345	–
Interaction	**0.000**		0.847	

Probability (*p*) values were calculated for treatment and treatment-time interaction effects. Tukey’s HSD *post hoc* results represent mean treatment values ordered from largest to smallest and significant differences (>) at α = 0.05, for urea (U), nitrate (NO), ammonium (NH), and the control (C). If a treatment falls on both sides of a “>” this indicates no significant difference from the treatments on either side. All phytoplankton biomass (mg L^−1^) data, but not chlorophyll *a* concentrations (µg L^−1^), were log_10_(x+1) transformed prior to analysis to meet assumptions of normality. Probabilities were not corrected for number of comparisons. See Methods for additional information.

**Figure 3:**
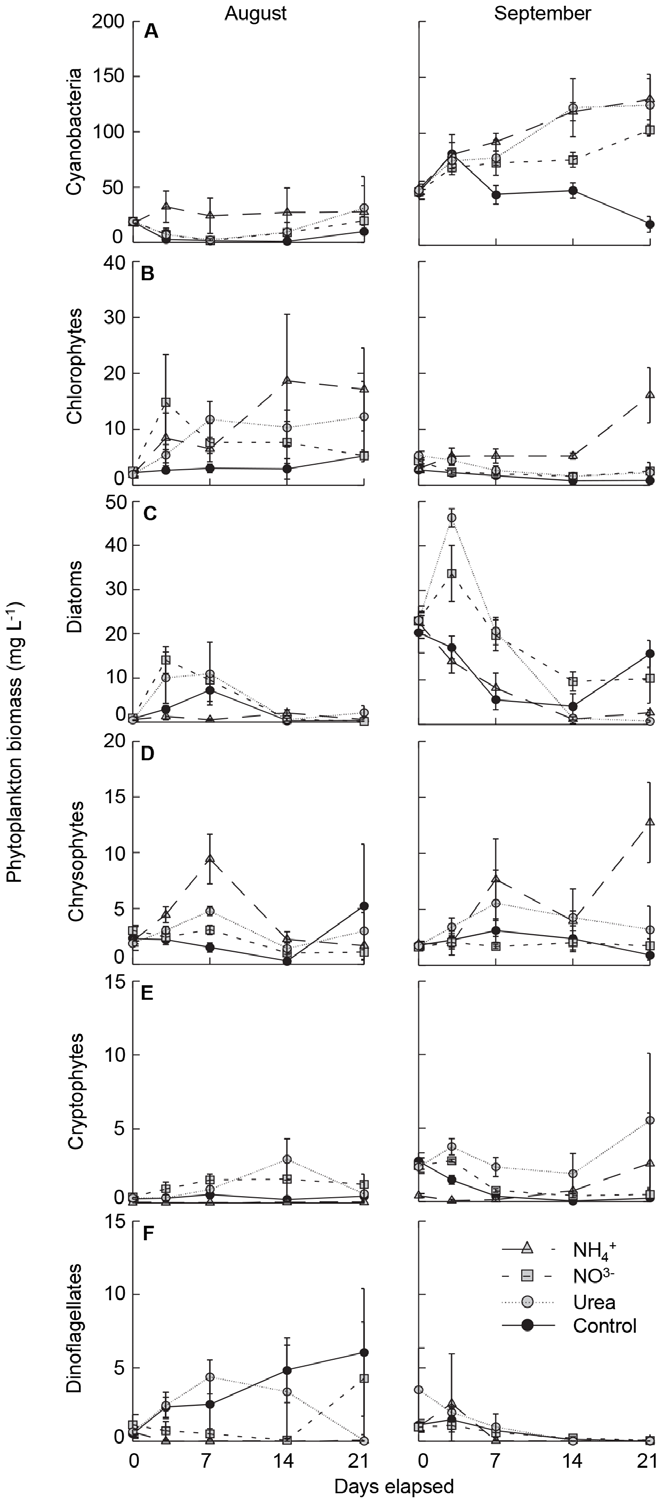
Biomass responses of major phytoplankton groups to fertilisation with nitrogen in mesocosms conducted in August and September. Algal groups (mg wet mass L^−1^) include; a) cyanobacteria, b) chlorophytes, c) diatoms, d) chrysophytes, e) cryptophytes and f ) dinoflagellates. Symbols represent mean and standard errors (± SE, *n* = 3) for each of the nitrogen treatments, included amendments with NH_4_^+^ (shaded triangle, coarse dashed line), NO_3_^−^ (shaded square, medium dashed line) and urea (shaded circle, fine dashed line), as well as unamended (control) mesocosms (solid circle, solid line).

The biomass response of algal divisions varied substantially with both phylogenetic group and chemical form of added N (Fig. 3, Table 1). In particular, abundance of cyanobacteria (Fig. 3a), chlorophytes (Fig. 3b) and chrysophytes (Fig. 3d) increased 400–800% following fertilisation with NH_4_^+^ and secondarily urea, changes which were significant during September (*p*_treatment_ <0.005), but only marginally so during August (0.05<*p*_treatment_ <0.11) due to high variability among NH_4_^+^ enclosures. In contrast, biomass of diatoms (Fig. 3c) and cryptophytes (Fig. 3e) increased ∼300% and ∼600% in treatments receiving NO_3_^−^ and urea, respectively, but were suppressed by addition of NH_4_^+^ (*p*_treatment_ <0.025) (Table 1). Finally, dinoflagellates were ∼500% more abundant in control mesocosms and those receiving urea during August relative to enclosures fertilised with NH_4_^+^ or NO_3_^−^ (*p*_treatment_ = 0.009), but showed little response to N additions during the September experiment (Fig. 3f).

### Species and Genus Response to N fertilisation

**Figure 4:**
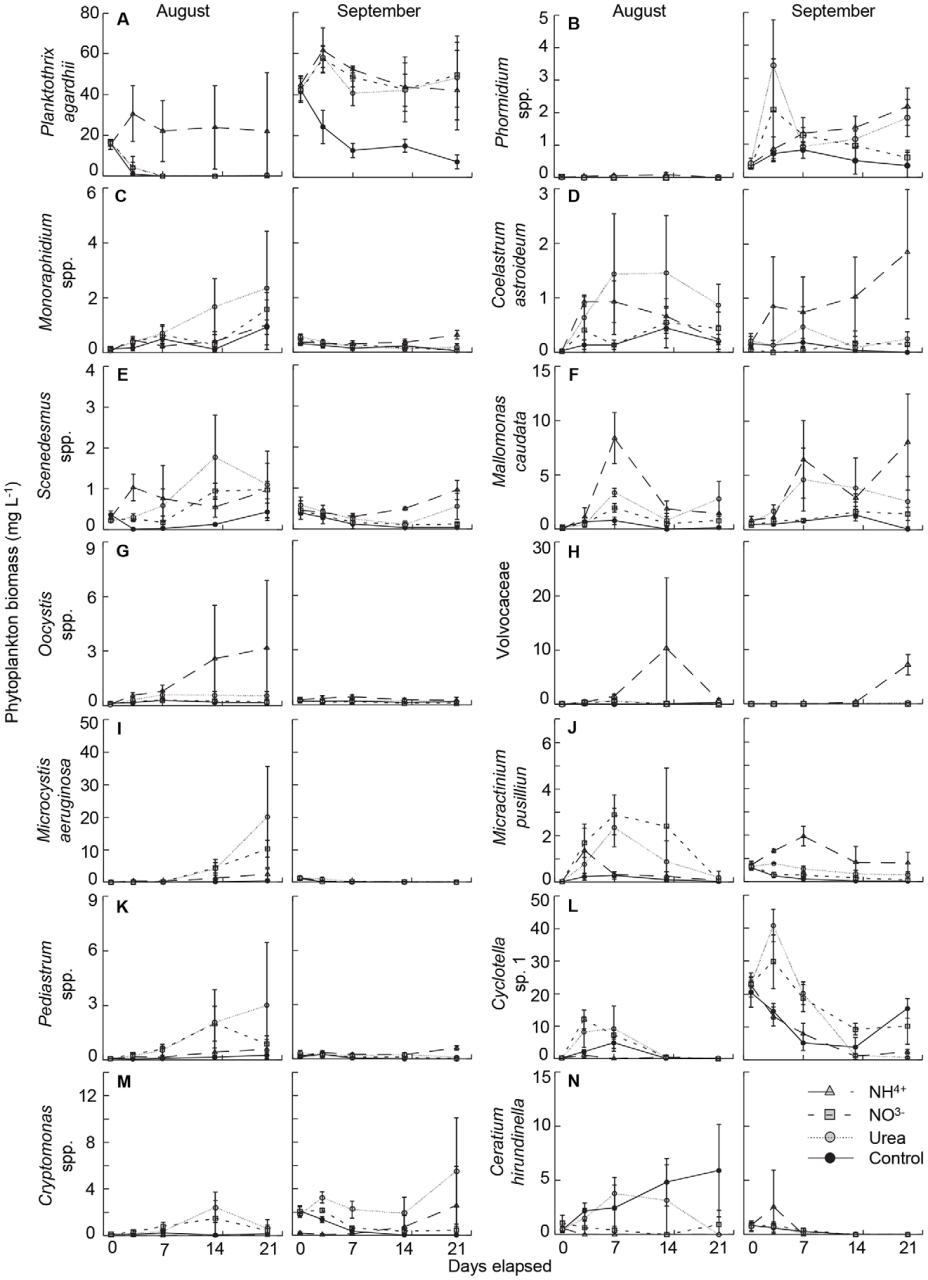
Biomass responses of important phytoplankton species to fertilisation with nitrogen in mesocosms conducted in Augusts and September. Biomass presented as mg wet mass L^−1^. Symbols represent mean and standard errors (± SE, *n* = 3) for each of the nitrogen treatments, including addition of NH_4_^+^ (shaded triangle, coarse dashed line), NO_3_^−^ (shaded square, medium dashed line) and urea (shaded circle, fine dashed line), as well as unamended (control) mesocosms (solid circle, solid line).

Phytoplankton assemblages in mesocosms consisted of 144 phytoplankton taxa, of which 51 responded significantly to N treatments (Table S1), 45 exhibited an increase in biomass following N fertilisation, and 14 had a wet-weight biomass of more than 1 mg L^−1^ on at least one sampling date (Fig. 4, Table 2). Overall, the biomass-dominant species exhibited distinctive responses to specific N compounds. For example, fertilisation with NH_4_^+^ consistently increased growth of *Planktothrix agardhii* (Fig. 4a), a species which accounted for up to 60% of total biomass, some members of the family Volvocaceae (*Pandorina morum*, *Pledorina illinoisensis*, *Gonium pectorale*) (Fig. 4h) (up to 50% of biomass) and *Oocystis* spp. (Fig. 4g) (∼10% of biomass). Similarly some algae were stimulated by both forms of chemically-reduced N, including *Phormidium* spp., *Monoraphidium* spp., *Scenedesmus* spp., *Coelastrum astroideum* and *Mallomonas caudata* (Fig. 4b–f). In contrast, species from the genera *Cyclotella* (Fig. 4l) and *Cryptomonas* (Fig. 4m) (10–40% of biomass) were stimulated more by NO_3_^−^ and urea than by NH_4_^+^, while the dinoflagellate *Ceratium hirundinella* (Fig. 4n) (25–50% of biomass) and rare diazotrophic cyanobacteria (*Aphanizomenon flos-aquae*, *Anabaena viguieri*, *Anabaena* spp.) grew poorly in fertilised enclosures. Finally, some abundant algae (*Microcystis aeruginosa*, *Micractinium pusillum*, *Pediastrum* spp.) (Figs 4i, j, k) (10–40% of biomass) showed inconsistent responses to fertilisation, with highest biomass recorded following amendment with NO_3_^−^ or urea in August, and with NH_4_^+^ during September.

**Table 2:** Repeated-measures analysis of variance for the response of selected phytoplankton taxa to added nitrogen.

Response variable	August		September	
	*p*	*Post hoc*	*p*	*Post hoc*
*Planktothrix agardhii*				
Treatment	**0.003**	NH>U, NO, C	**0.000**	NH, NO, U>C
Interaction	**0.015**		**0.001**	
*Phormidium* spp.				
Treatment	0.103	–	**0.014**	U, NH, NO>NO, C
Interaction	0.101		**0.010**	
*Monoraphidium* spp.				
Treatment	0.174	–	**0.041**	NH, U, NO>U, NO, C
Interaction	0.524		**0.002**	
*Coelastrum astroideum*				
Treatment	0.056	–	0.117	–
Interaction	0.364		0.222	
*Scenedesmus* spp.				
Treatment	**0.013**	U, NH, NO>NO, C	**0.011**	NH, U>U, NO, C
Interaction	0.144		**0.002**	
*Mallomonas caudata*				
Treatment	**0.001**	NH, U>U, NO>NO, C	**0.001**	NH,U>U, NO>NO, C
Interaction	**0.006**		**0.046**	
*Oocystis* spp.				
Treatment	0.244	–	**0.010**	NH, U>U, C, NO
Interaction	0.523		0.746	
Volvocaceae				
Treatment	0.059	–	**0.000**	NH>U, C, NO
Interaction	0.247		**0.000**	
*Microcystis aeruginosa*				
Treatment	0.122	–	0.092	–
Interaction	**0.003**		**0.046**	
*Micractinium pusillum*				
Treatment	**0.047**	NO, U, NH>U, NH, C	**0.000**	NH>U>NO, C
Interaction	**0.024**		0.063	
*Pediastrum* spp.				
Treatment	0.249	–	**0.004**	NH, U>U, NO>NO, C
Interaction	0.262		**0.000**	
*Cyclotella* spp.				
Treatment	**0.013**	NO, U, C>C, NH	**0.012**	NO, U, C>U, C, NH
Interaction	**0.002**		**0.000**	
*Cryptomonas* spp.				
Treatment	0.084	–	**0.023**	U, NO>NO, C, NH
Interaction	**0.018**		**0.002**	
*Ceratium hirundinella*				
Treatment	**0.003**	C, U>U, NO>NO, NH	0.906	–
Interaction	**0.006**		0.995	

Probability (*p*) values were calculated for treatment and treatment-time interaction effects. Tukey’s HSD *post hoc* results represent mean treatment values ordered from largest to smallest and significant differences (>) at α = 0.05, for urea (U), nitrate (NO), ammonium (NH), and the control (C). If a treatment falls on both sides of a “>” this indicates no significant difference from the treatments on either side. All phytoplankton biomass (mg L^−1^) data were log_10_(x+1??transformed prior to analysis to meet assumptions of normality. Probabilities were not corrected for number of comparisons.

### Influence of Taxonomic Resolution on Interpretation of N effects

**Figure 5:**
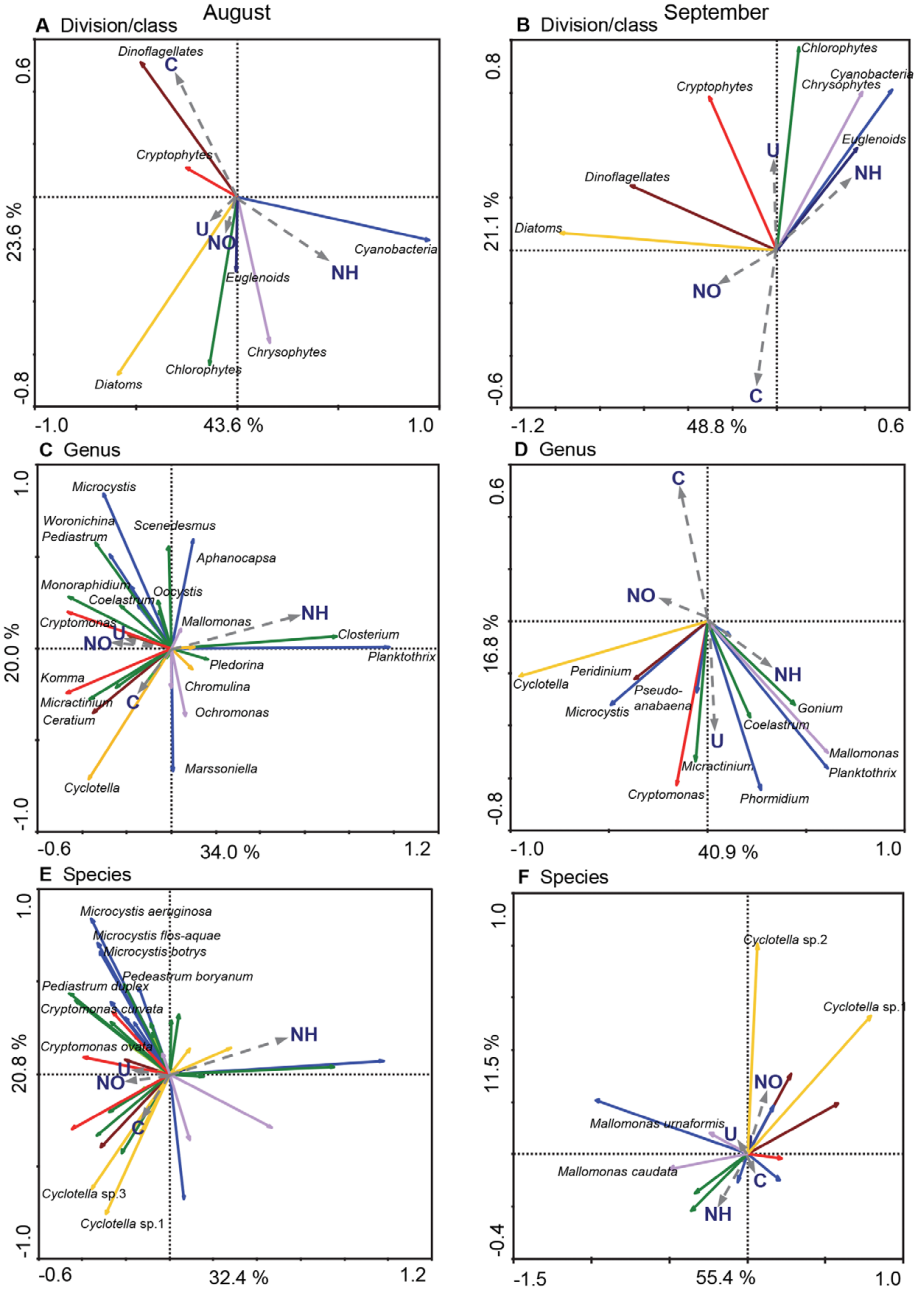
Principal component analysis of experimental phytoplankton assemblages at the a) division, b) genus, and c) species level of taxonomic resolution. Genera and species were selected if their cumulative biomass over the course of each experiment was more than 1% of the total for any of the 12 enclosures. Algal densities were log_10_(x +1)-transformed as needed, and categorical nitrogen treatments (e.g.,+or – urea) were included as passive variables. All samples were included in each PCA; however, to simplify presentation, sample ordination points are not presented and only select taxa are identified. Coloured arrows indicate cyanobacteria (blue), chlorophytes (green), cryptophytes (red), diatoms (yellow), dinoflagellates (brown), and chrysophytes (purple). Proportion of total variation explained by first (x) and second (y) principle axes are presented.

PCA captured 53.2–69.9% of variation in phytoplankton community composition on the first two ordination axes when phytoplankton was resolved to level of division, genus or species (Fig. 5). Total explained variation decreased only slightly with increased taxonomic resolution. In general, axis 1 explained more variance in the September experiment (40.9–55.4%) than during August trials (32.4–43.6%). During August, axis 1 was associated positively with NH_4_^+^ at all taxonomic levels and negatively with NO_3_^−^ and urea for PCAs with genus or species resolution, while axis 2 was associated most strongly with control mesocosms at all taxonomic resolutions. During September, axis 1 was correlated positively with NH_4_^+^ treatments and negatively with NO_3_^−^ treatments at the division and genus levels, but was not related linearly to any N treatment in the PCA of algal species. In contrast, axis 2 was associated positively with the urea, control, and NO_3_^−^ treatments at the division, genus and species levels, respectively, and negatively with the control, urea, and NH_4_^+^ trials at the division, genus and species levels, respectively.

PCA of algal divisions confirmed that total cyanobacterial biomass was associated positively with NH_4_^+^ treatments in both experiments, while that of chrysophytes and euglenoids responded positively to NH_4_^+^ in September alone (Figs. 5a, b). In contrast, chlorophyte, cryptophyte and diatom abundances were elevated in mesocosms treated with urea and NO_3_^−^ during August, but NO_3_^−^ (diatoms) or urea alone (chlorophytes and cryptophytes) during September. Finally, dinoflagellate abundance was associated mainly with control enclosures in August, but not during September.

Multivariate analysis at a finer taxonomic resolution demonstrated that genera within algal divisions often exhibited individualistic responses to N treatments (Fig. 5c, d). Among cyanobacteria, *Planktothrix* was strongly associated with NH_4_^+^ treatments in both experiments, whereas *Microcystis* was abundant in enclosures amended with urea and NO_3_^−^ only during August, and *Phormidium* was abundant in urea treatments during September. Similarly, the chlorophytes *Closterium* and *Pleodorina* were associated positively with NH_4_^+^ treatments during August; however, *Micractinium* and *Coelastrum* were common in urea amendments during September, while *Monoraphidium* and *Pediastrum* were associated with urea treatments in both experiments and with NO_3_^−^ in during August. Divergent responses were also recorded for cryptophytes genera, with *Cryptomonas* being associated positively with NO_3_^−^ fertilisation during August and urea treatments in both months, and *Komma* exhibiting elevated abundance within control enclosures during August. Finally, the chrysophyte *Mallomonas* exhibited the fundamentally different responses to those of *Chromulina* and *Ochromonas*.

PCA of common algal species (>1% of biomass) revealed low variability among congeneric taxa in response to N treatments (Figs. 5e, f). For example, *Microcystis aeruginosa*, *M. botrys* and *M. flos-aquae* were all associated weakly with urea and NO_3_^−^ treatments during August. Similarly *Cryptomonas ovata* and *C. erosa*, as well as *Pediastrum duplex* and *P. boryanum*, responded positively to NO_3_^−^ and urea treatments, whereas *Synedra acus* and *S. ulna* were common in NH_4_^+^ treatments. Congruent ordination of congeneric species was also evident for members of the genera *Cyclotella* and *Mallomonas*.

### Comparison of Microscopy and HPLC

Changes in phytoplankton biomass determined by microscopy were correlated significantly (*p*_treatment_ <0.005) with variations in concentrations of taxonomically-diagnostic pigments for all major algal groups (Table 3). The strongest correlations were observed for Chl *b*-chlorophytes (*r* ^2^ = 0.42–0.63), myxoxanthophyll-colonial cyanobacteria (*r* ^2^ = 0.31–0.52), and fucoxanthin-siliceous algae (*r* ^2^ = 0.29–0.46), although significant linear relationships were recorded for alloxanthin-cryptophytes (*r* ^2^ = 0.18–0.28) and peridinin-dinoflagellates (*r* ^2^ = 0.13–0.36).

**Table 3:** Least-squares regression analysis of the linear relationship between microscopic and chromatographic estimates of phytoplankton abundance.

Model		August		September	
Pigment	Algal group	*r* ^2^	*p*	*r* ^2^	*p*
Chlorophyll *a*	Total biomass	0.641	0.000	0.418	0.000
Myxoxanthophyll	Colonial cyanobacteria	0.313	0.000	0.523	0.000
Chlorophyll *b*	Chlorophytes	0.630	0.000	0.416	0.000
Fucoxanthin	Chrysophytes and diatoms	0.292	0.000	0.458	0.000
Alloxanthin	Cryptophytes	0.280	0.000	0.181	0.000
Peridinin	Dinoflagellates	0.355	0.000	0.129	0.005

Phytoplankton biomass was measured by microscopy, while concentrations of taxonomically-diagnostic biomarker pigments were analysed by spectrophotometry (chlorophyll *a*) and high performance liquid chromatography (all other pigments). Data were log_10_(x+1) transformed prior to analysis (*df* = 58). Algal biomass was summed according to distribution of indicator pigments prior to statistical analysis.

**Figure 6:**
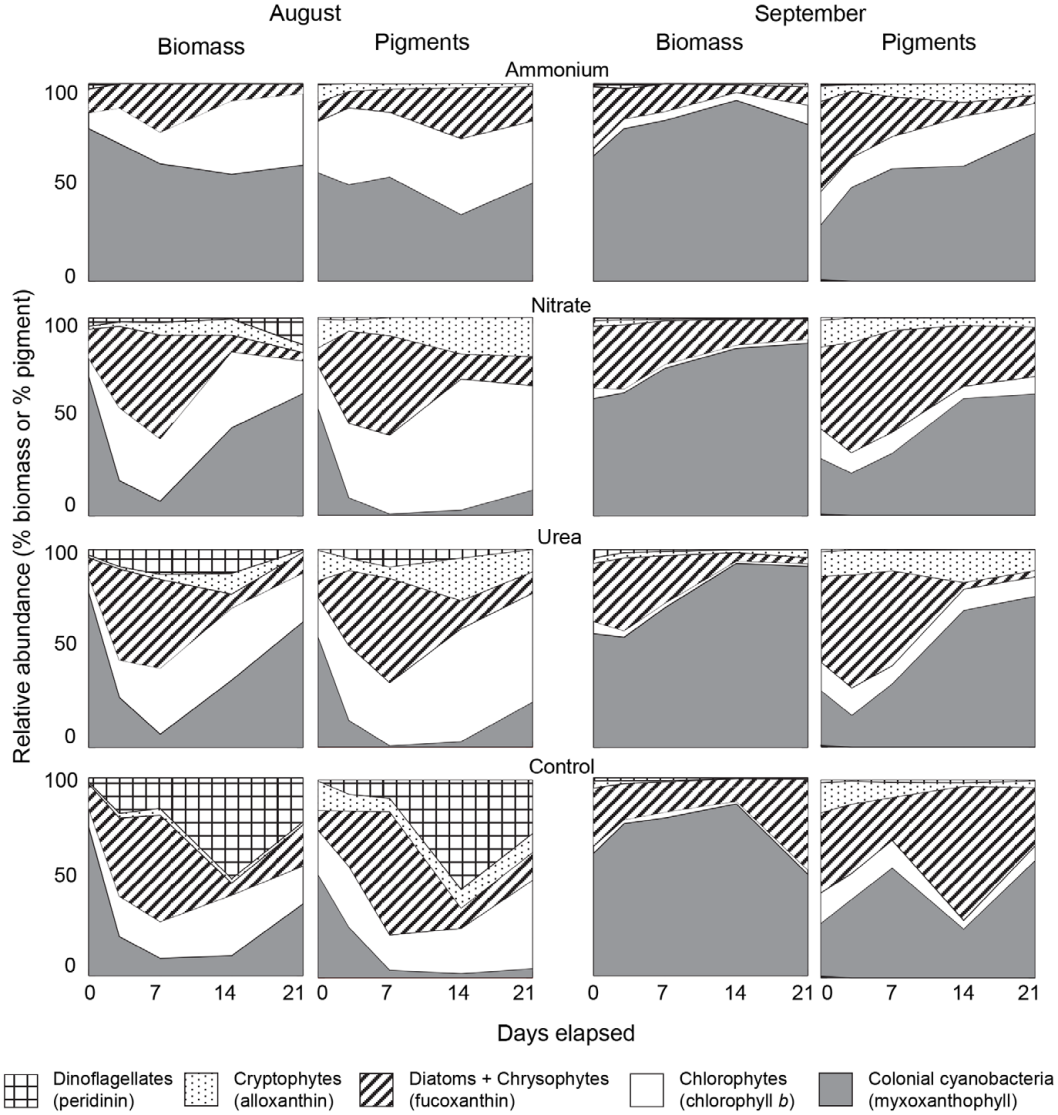
Mean relative abundance of the major phytoplankton groups in the mesocosms subject to addition of nitrogen. Treatment include addition of ammonium, nitrate, urea and no nitrogen (control) (n = 3). Phytoplankton abundance was determined by microscopic enumeration of biomass and by high performance liquid chromatography of algal pigments in experiments conducted during August and September 2008. Algal groups (and pigments) include dinoflagellates (peridinin), cryptophytes (alloxanthin), diatoms and chrysophytes (fuoxanthin), chlorophytes (chlorophyll *b*) and colonial cyanobacteria (myxoxanthophyll).

Patterns of temporal change in gross community composition (division level) were also very similar when analysed by microscopy or HPLC (Fig. 6). Overall, HPLC analysis tended to underestimate cyanobacterial contributions to the phytoplankton community, whereas chlorophytes and cryptophytes composed a greater fraction of total abundance when based on biomarker pigments. Similarly, siliceous algae (diatoms+chrysophytes) were slightly overrepresented by pigment analysis in the September experiment, but not during trials conducted in August (Fig. 5e, f).

## Discussion

Three-fold expansion of agricultural fertilisation [1], exponential growth of cities [2], high infrastructure costs to eliminate waste N [63], and scientific debate concerning the role of N in eutrophication [13], [28] have combined to prolong N pollution and degrade some freshwater ecosystems [11], [64]. In part, uncertainty over best management practices may arise because we do not often distinguish clearly how algal response to N pollution differs among taxonomic groups, whether the response of individual taxa varies with the chemical form of N, or how differences in analytical approach (microscopy, pigment biomarkers) affect the interpretation of N effects on water quality. Analysis of experiments herein demonstrates that growth of >30% of phytoplankton species in eutrophic lake waters was stimulated significantly by N fertilisation (Table S1), and that the differential response of algal divisions to each chemical form of N (Fig. 3) resulted from distinct responses of algal genera rather than from unique responses of congeneric species (Fig. 5) consistent with [65]. Specifically, fertilisation with NH_4_^+^ increased total algal abundance ∼350% (Fig. 2) and cyanobacterial biomass over 500% (Fig. 3a) because *Planktothrix agardhii* accounted for up to 60% of phytoplankton biomass and its growth increased nearly six-fold following addition of chemically-reduced N (Fig. 4a). In contrast, *Microcystis aeruginosa* responded mainly to NO_3_^−^ addition, particularly when waters were warm [20], [66]. These experiments also documented significant correlations between microscopic- and HPLC-based estimates of algal abundance (Table 3) consistent with previous calibration exercises [45], [46], [67]. As well, the highly similar patterns of temporal change in assemblage composition (Fig. 6) infer that pigment-based investigations accurately represent how N can degrade lake ecosystems [6], [11], [20], [25]. When considered in the context of previous laboratory [17], [18], mesocosm [19], [20], whole-lake [21], [68], catchment [11], [23], [24] and palaeolimnological studies [6], [11], the present analysis confirms that pollution with diverse forms of N can degrade P-rich lakes by promoting toxic cyanobacteria such as *Microcystis* and *Planktothrix*, but shows that there is no unique response of ‘cyanobacteria’ as has been suggested in management studies [28], [48].

### Response of Predominant Phytoplankton to N fertilisation

Addition of NH_4_^+^ to P-rich lake water favoured a community composed predominantly of nondiazotrophic *Planktothrix agardhii*, a taxon known to produce high levels of microcystin [69], [70]. Elevated growth of *P. agardhii* likely reflects its lower K*m* for reduced N [33], higher maximum uptake rates [71] relative to other taxa [38], and the general preference of cyanobacteria for chemically-reduced forms of N [17], [36]. In addition, low energetic requirements for NH_4_^+^ assimilation would have allowed *P. agardhii* to maintain a high biomass and shade out competitors, thereby perpetuating a competitive advantage for this low-light-adapted taxon [72], [73]. Continued dominance of *P. agardhii* into mid-September was also consistent with algal phenologies seen in other unstratified lakes [6] and with this species’ higher tolerance to low temperatures relative to that of *Microcystis* [66], [74]. Such selective stimulation of *Planktothrix* growth by N can also result in five- to 10-fold increases in water-column concentrations of microcystin [20], [25], as transcription of toxin synthesis genes is also up-regulated by N assimilation [75].

Nitrate amendment favoured initial growth of large centric diatoms such as *Cyclotella* spp. (Figs. 3c, 4l), before giving way to colonial *Microcystis* spp. during August (Fig. 4i). As shown elsewhere, members of the genus *Cyclotella* are often abundant in NO_3_^–^rich eutrophic waters [37], [38], [76], possibly because they exhibit higher affinity for NO_3_^−^ [77] and non-saturating uptake kinetics for that compound [39]. However, under conditions in which their growth becomes limited by the supply of P, Si or light [37], [78], diatoms can be replaced by dense blooms of slower-growing *Microcystis* spp. [43]. Members of this latter genus can exhibit relatively low K*m* [33] and high V_max_ [79] for NO_3_^−^ under *in vitro* conditions, are capable of substantial storage of P [10], and use vertical migration to optimize energy receipt [80]. However, we recommend caution when interpreting the mechanisms underlying these rapid (<7 day) changes in algal composition both because our experimental design may favour limitation of diatom growth by Si [20], and because *Microcystis* abundance is also suppressed by water temperatures less than 20°C [66], [81]. Consequently, although our mesocosms are suitable for evaluation of many pelagic processes [20], we suggest that further *in situ* experimentation be conducted to evaluate controls of diatom abundance, including use of mesocosms which include benthic habitats.

Addition of urea to eutrophic environments stimulated the growth of many phytoplankton taxa (Table S1; Figs 4, 5), similar to findings from earlier laboratory [18], [30], [82] and field studies [20], [83]. Preference for urea as a N source may be widespread among algae because this compound enters cells by passive diffusion or light-independent transporters [84], transports two NH_4_^+^ for every molecule acquired [85], is assimilated into organic matter without intracellular chemical reduction [36], and releases CO_2_ following assimilation, partly reducing the need for active uptake of HCO_3_^−^ at high pH [86]. In fact, energetic costs for assimilation of chemically-reduced N species are less than half that associated with atmospheric N or dissolved NO_3_^−^ [17], consistent with our observation that total algal biomass was ∼1.5-fold higher when phytoplankton received NH_4_^+^ or urea than when NO_3_^−^ was added (Fig. 2). However, despite expected energetic benefits of urea and NH_4_^+^, the wide variety of phytoplankton species response to different chemical forms of N (Fig. 3, 4) demonstrates that factors other than simple energetic costs of assimilation must also influence algal response to N, including temperature [20], [25], nutrient co-limitation [31], [37], or cellular stoichiometry [77], [87].

Interestingly, all forms of N amendment increased growth of chlorophyte algae (Figs 3b, 5e), including 26 of 69 taxa (Table S1). Chlorophytes are sometimes associated with N-enriched eutrophic environments [19], particularly those of shallow lakes where light may penetrate to benthic substrates [21], [34]. In general, green algae are thought to have high light requirements [32] and, if sufficiently illuminated, are competitive with other phytoplankton due to high rates of cell division and diverse mechanisms of N assimilation [88]. However, the lack of consistent response among green algal genera to individual forms of N (Fig. 4) suggests that pronouncements of division-level response of chlorophytes to pollution with N are premature [48], and that further research is needed to evaluate the complex relationships between lake depth, irradiance regime, and N influx as factors regulating growth of chlorophyte algae [34].

### Effects of Taxonomic Resolution on Interpretation of N effects

Comparison among PCAs (Fig. 5) revealed that generalisations concerning algal response to N addition depended on the taxonomic resolution of the microscopic analysis. Within the algal divisions, genera exhibited high variability in response to added N (Fig. 5c, d), likely reflecting substantial differences in morphology, growth capabilities and nutrient uptake kinetics (see above). Given the wide range in K*m* and V_max_ observed in studies of isolated algae and natural communities [41], [89], [90], it has been inferred that variations among genera mainly reflect substantial differences in colony size (µm-mm) and cell volume [91] for many divisions [42], [43]. In contrast, similar responses of closely-related species to N amendments (Fig. 5e, f) may occur because the fundamental niche of individual taxa has not yet diverged from shared lineages [65], [90], [92], recent speciation has conserved important morphological and physiological traits [93] which control of nutrient use [91], or local ecological interactions have already selected for congeneric species with similar ecological attributes. However, irrespective of the precise mechanism, the highly similar responses of congenetic species to added N (e.g., *Microcystis aeruginosa*, *M*. *flos-aquae*, *M*. *botrys*) combined with the low congruence of genera within a given division (e.g., *Anabaena*, *Planktothrix*, *Microcystis, Aphanizomenon*) (Table S1), suggests that important ecological insights on the role of N in lake eutrophication may be obtained without laborious identification of all phytoplankton to species identity [92], assuming patterns observed herein generalise well to other lakes.

### Effects of Analytical Method on Interpretation of N effects

Empirical (Fig. 6) and statistical (Table 3) comparison of phytoplankton community composition derived from phytoplankton analysis by microscopy and HPLC demonstrates that changes in concentrations of biomarker pigments were influenced mainly by variation in abundance of the predominant algae rather than by the precise species composition of phytoplankton, irradiance regime or nutrient availability [46], [59], [67]. Although cellular pigment content (e.g., Chl *a* cell^−1^) increases directly with N availability and inversely with irradiance levels in the laboratory [47], cellular quotas did not appear to be the main factor regulating HPLC-based inference of algal abundance and pigment-biomass correlations were of a similar magnitude to those quantified in earlier studies [46], [59], [67]. That said, we recognize that the uneven distribution of some indicator pigments among genera within some functional groups (e.g., aphanizophyll among N-fixing cyanobacteria) [59] may complicate pigment-based interpretations of algal community change unless augmented by microscopic enumeration of critical samples [6]. Fortunately, the generally high correspondence between microscopic and chromatographic analyses (Fig. 6), combined with the widespread use of similar HPLC and microscopic protocols [59], [94], suggests that previous investigations based solely on biomarker pigments have provided robust and reliable information about N effects on aquatic ecosystems.

Despite evidence that growth of 45 phytoplankton species (31.5% of taxa) was stimulated by addition of dissolved N to P-rich lake waters (Table S1, Fig. 5), further research is required to evaluate the reasons for limited response of the remaining 99 taxa, including 6 species whose growth was suppressed by N amendments (Fig. 4n). For example, bottle bioassays of the nutritional status of algae in Wascana and other regional lakes [20], [25] suggests that addition of NH_4_^+^ during May can suppress primary production when phytoplankton are composed mainly of diatoms, chrysophytes and cryptophytes [49] and that additional seasonal analysis of N effects is warranted. Further, improved understanding of the effects of N influx on the relative proportion of chlorophytes and non-N-fixing cyanobacteria is also needed [34], and would benefit from analysis of gross community changes along a gradient of N addition, as well as from comparisons of chlorophyte response in lakes of differing depths or water clarity (see above). Taken together, such refinement of our understanding of the phytoplankton-specific responses to N pollution may provide the best means of averting future damage to aquatic ecosystems arising from doubled N influx by 2050.

## Supporting Information

Table S1 Phytoplankton taxa and response to nitrogen fertilisation in August and September experiments.
(DOC)
