# Supplementary material for: Phytoplankton-Specific Response to Enrichment of Phosphorus-Rich Surface Waters with Ammonium, Nitrate, and Urea
Source: PLoS One. 2013 Jan 17;8(1):e53277. doi: 10.1371/journal.pone.0053277 (PMC3547936; doi:10.1371/journal.pone.0053277)
Supplement: Table S1 — Phytoplankton taxa and response to nitrogen fertilisation in August and September experiments. (DOC) [file pone.0053277.s001.doc]

Supporting Information Table S1. **Phytoplankton taxa and response to nitrogen fertilisation in August and September experiments.** Symbols represent taxa showing significant positive (+) or negative (-) effects of nitrogen treatments, including addition of ammonium (NH_4_^+^), nitrate (NO_3_^-^) or urea (U) relative to unamended control mesocosms. Statistical significance was analysed using repeated measures analysis of variance (RM-ANOVA) and Tukey’s Honestly Significant Difference *post hoc* test, but was not corrected for the number of comparisons. Responses were significant at *p* < 0.1 (+, -), *p* < 0.05 (++, --), *p* < 0.01 (+++, ---) or *p* < 0.001 (++++, ----). Multiple species are identified as spp., whereas unknown species are identified as sp. See main text for taxonomic references and additional methodological details.

| Taxon | Code |  | August | |  |  | Sept. |  | |
| --- | --- | --- | --- | --- | --- | --- | --- | --- | --- |
|  |  | NH_4_^+^ | NO_3_^-^ | | U | NH_4_^+^ | NO_3_^-^ | U | |
| CYANOPHYTA |  | + |  | |  | ++++ | +++ | ++++ | |
|  |  |  |  | |  |  |  |  | |
| *Anabaena* sp. | CY1 |  |  | |  |  |  |  | |
| *Anabaena viguieri* Denis & Frémy | CY2 |  |  | |  | - - - | - - - |  | |
| *Anabaenopsis circularis* (G.S.West) Woloszynska & Miller | CY3 | ++ |  | |  |  |  |  | |
| *Aphanizomenon flos-aquae* Ralfs ex Bornet & Flahault | CY4 |  |  | |  |  |  | - | |
| *Aphanocapsa grevillei* (Hassall) Rabenhorst | CY5 |  |  | |  |  |  |  | |
| *Aphanocapsa pulchra* (Kützing) Rabenhorst | CY6 |  |  | |  |  |  |  | |
| *Aphanocapsa* spp. | CY7 |  |  | |  |  |  |  | |
| *Aphanothaceae* sp.1 | CY8 |  |  | |  |  |  |  | |
| *Arthrospira* sp. | CY9 |  |  | |  |  |  |  | |
| *Chroococcus limneticus* Lemmermann | CY10 |  |  | |  |  |  |  | |
| *Chrocooccus minutus* (Kützing) Nägeli | CY11 |  |  | |  |  |  |  | |
| *Coelosphaerium kuetzingianum* Nägeli | CY12 |  |  | |  |  |  |  | |
| *Eucapsis* sp. 1 | CY13 |  | + | |  |  |  |  | |
| *Marssoniella elegans* Lemmermann | CY14 |  |  | |  |  |  |  | |
| *Merismopedia tenuissima* Lemmermann | CY15 |  |  | |  |  |  | +++ | |
| *Merismopedia minima* Beck | CY16 |  |  | |  |  |  | ++ | |
| *Microcystis aeruginosa* (Kützing) Kützing | CY17 |  |  | |  |  |  | + | |
| *Microcystis botrys* Teiling | CY18 |  |  | |  |  |  |  | |
| *Microcystis flos-aquae* (Wittrock) Kirchner | CY19 |  | ++ | |  |  |  |  | |
| *Microcystis microcystiformis* (F.Hindak) A.M.T. Joosten | CY20 |  |  | |  |  |  |  | |
| *Microcystis* spp. | CY21 |  |  | |  |  |  |  | |
| *Microcystis wesenbergii* (Komárek) Komárek | CY22 |  |  | |  |  |  |  | |
| *Oscillatoria* sp.1 | CY23 |  |  | |  |  |  |  | |
| *Phormidium foveolarum* Montagne ex Gomont | CY24 |  |  | |  | ++ |  | +++ | |
| *Phormidium* sp.1 | CY25 |  |  | |  |  |  |  | |
| *Phormidium* spp. | CY26 |  |  | |  |  |  |  | |
| *Phormidium tenue* (C.Agardh ex Gomont) Anagnostidis & Komárek | CY27 |  |  | |  |  |  |  | |
| *Planktothrix agardhii* (Gomont) Anagnostidis & Komárek | CY28 | +++ |  | |  | +++ | +++ | +++ | |
| *Planktothrix mougeotii* (Kützing ex Forti) Suda, Watanabe, Otsuka, Mahakahant, Yongmanichtai, Nopartnaraporn, Liu & Day | CY29 |  |  | |  |  |  |  | |
| *Pseudoanabaena catenata* Lauterborn | CY30 |  |  | |  |  |  |  | |
| *Pseudoanabaena* spp. | CY31 |  |  | |  |  |  |  | |
| *Radiocystis* sp.1 | CY32 |  |  | |  |  |  |  | |
| *Senechrocystis* sp.1 | CY33 |  |  | |  |  |  |  | |
| *Snowella* sp.1 | CY34 |  |  | |  |  |  |  | |
| *Spirulina* sp.1 | CY35 |  |  | |  |  |  |  | |
| *Synechococcus* sp.1 | CY36 |  |  | |  |  |  |  | |
| *Woronichinia delicatula* (Skuja) Komárek & Hindák | CY37 |  |  | |  |  |  |  | |
| *Woronichinia robusta* (Skuja) Komárek & Hindák | CY38 |  |  | |  |  |  |  | |
|  |  |  |  | |  |  |  |  | |
| DINOFLAGELLATA |  | - - - |  | |  |  |  |  | |
|  |  |  |  | |  |  |  |  | |
| *Ceratium hirundinella* (O.F.Müller) Dujardin | DN1 | - - - | - - | |  |  |  |  | |
| *Gymnodinium* sp.1 | DN2 |  |  | |  |  |  |  | |
| *Gymnodinium* sp.2 | DN3 |  |  | |  |  |  |  | |
| *Gymnodinium* sp.3 | DN4 |  |  | |  |  |  |  | |
| *Peridinium* spp. | DN5 |  |  | |  |  |  |  | |
|  |  |  |  | |  |  |  |  | |
| CHLOROPHYTA |  | ++ |  | | + | +++ |  | + | |
|  |  |  |  | |  |  |  |  | |
| *Actinastrum gracillimum* Smith | CL1 |  |  | |  |  |  |  | |
| *Actinastrum hantzschii* Lagerheim | CL2 |  |  | |  |  |  |  | |
| *Ankistrodesmus falcatus* (Corda) Ralfs | CL3 |  |  | |  |  |  |  | |
| *Asterococcus limneticus* G.M. Smith | CL4 |  |  | |  |  |  |  | |
| *Carteria* sp.1 | CL5 |  |  | |  | - |  | - | |
| *Characium* sp.1 | CL6 |  |  | |  |  |  |  | |
| *Chlamydomonas globosa* J.W.Snow | CL7 |  |  | |  |  |  |  | |
| *Chlamydomonas* spp. | CL8 |  |  | |  | +++ |  |  | |
| *Chlorella vulgaris* Beyerinck [Beijerinck] | CL9 |  |  | |  |  |  |  | |
| *Closteriopsis acicularis* (Chodat) J.H.Belcher & Swale | CL10 |  |  | |  |  |  |  | |
| *Closterium acutum* Brébisson | CL11 | +++ |  | |  |  |  |  | |
| *Closterium dianae* Ehrenberg ex Ralfs | CL12 |  |  | |  |  |  |  | |
| *Closterium strigosum* Brébisson | CL13 |  |  | |  |  |  |  | |
| *Coelastrum astroideum* De Notaris | CL14 |  |  | | + |  |  |  | |
| *Coelastrum microporum* Nägeli | CL15 |  |  | |  |  |  |  | |
| *Coelastrum sphaericum* Nägeli | CL16 |  |  | |  |  |  |  | |
| *Coenocystis obtusa* Korshikov | CL17 |  |  | |  |  |  |  | |
| *Cosmarium* sp.1 | CL18 |  | + | |  |  |  |  | |
| *Diacanthos belenophorus* Korshikov | CL19 |  |  | |  | + |  |  | |
| *Dictyosphaerium pulchellum* H.C.Wood | CL20 |  |  | | + |  |  |  | |
| *Dictyosphaerium* spp. | CL21 |  |  | |  |  |  |  | |
| *Eudorina elegans* Ehrenberg | CL22 |  |  | |  |  |  |  | |
| *Golinkia radiate* Chodat | CL23 |  |  | |  |  |  |  | |
| *Goniochloris fallax* Fott | CL24 |  | ++ | |  |  |  |  | |
| *Gonium pectorale* O.F.Müller | CL25 |  |  | |  | ++++ |  |  | |
| *Keratococcus bicaudatus* (A.Braun ex Raenhorst) J.B.Petersen | CL26 |  |  | |  |  |  |  | |
| *Kirchneriella* sp.1 | CL27 |  |  | |  | +++ |  | ++ | |
| *Lagerheimia chodatii* C.Bernard | CL28 |  |  | |  |  |  |  | |
| *Lagerheimia subsalsa* Lemmermann | CL29 |  |  | |  |  |  |  | |
| *Lagerheimia wratislaviensis* Schröder | CL30 |  |  | |  |  |  |  | |
| *Micractinium pusillum* Fresenius | CL31 |  | ++ | |  | ++++ |  | ++++ | |
| *Micractinium quadrisetum* (Lemmermann) G.M.Smith | CL32 |  |  | |  |  |  |  | |
| *Monoraphidium arcuatum* (Korshikov) Hindák | CL33 |  |  | |  |  |  |  | |
| *Monoraphidium contortum* (Thuret) Komàrková-Legnerová | CL34 |  |  | |  | + |  |  | |
| *Monoraphidium convolutum* (Corda) Komàrková-Legnerová | CL35 |  |  | |  | ++++ |  | + | |
| *Monoraphidium irregulare* (G.M.Smith) Komàrková-Legnerová | CL36 |  |  | |  |  |  |  | |
| *Monoraphidium griffithii* (Berkeley) Komàrková-Legnerová | CL37 |  |  | |  | ++ |  |  | |
| *Monoraphidium minutum* (Nägeli) Komàrková-Legnerová | CL38 |  |  | |  |  |  |  | |
| *Oocystis* spp. | CL39 |  |  | |  | ++ |  |  | |
| *Pandorina morum* (O.F.Müller) Bory de Saint-Vincent | CL40 |  |  | |  | ++++ |  |  | |
| *Pediastrum boryanum* (Turpin) Meneghini | CL41 |  |  | |  | +++ |  | + | |
| *Pediastrum duplex* Meyen | CL42 |  |  | |  | + |  |  | |
| *Pleodorina illinoisensis* Kofoid | CL43 |  |  | |  | + |  |  | |
| *Polyedriopsis spinulosa* (Schmidle) Schmidle | CL44 |  |  | |  |  |  |  | |
| *Quadricula* sp.1 | CL45 |  |  | |  |  |  |  | |
| *Scenedesmus abundans* (O.Kirchner) Chodat | CL46 |  |  | | + |  |  | ++ | |
| *Scenedesmus acuminatus* (Lagerheim) Chodat | CL47 |  |  | |  | +++ |  |  | |
| *Scenedesmus bicaudatus* Dedusenko | CL48 |  |  | |  |  |  |  | |
| *Scenedesmus bijuga* (Turpin) Lagerheim | CL49 |  |  | |  |  |  | + | |
| *Scenedesmus dimorphus* (Turpin) Kützing | CL50 |  |  | |  |  |  |  | |
| *Scenedesmus falcatus* Chodat | CL51 |  |  | | ++ | +++ |  |  | |
| *Scenedesmus intermedius* Chodat | CL52 | ++ |  | |  |  |  |  | |
| *Scenedesmus aculeolatus* Reinsch | CL53 |  |  | |  |  |  |  | |
| *Scenedesmus obtusus* Meyen | CL54 |  |  | |  |  |  |  | |
| *Scenedesmus opoliensis* P.G.Richter | CL55 | ++ |  | |  | + |  | ++ | |
| *Scenedesmus quadricauda (communis)* E.Hegewald | CL56 | + |  | |  |  |  |  | |
| *Scenedesmus* spp. | CL57 |  |  | |  | + |  |  | |
| *Schroederia robusta* Korshikov | CL58 |  |  | |  |  |  |  | |
| *Schroederia setigera* (Schröder) Lemmermann | CL60 |  |  | |  |  |  |  | |
| *Sphaerellopsis spiralis* J.H.Belcher & Swale | CL61 |  |  | |  |  |  |  | |
| *Sphaerocystis planktonica* (Koršikov) Bourrelly | CL62 | - - | - - | | - - |  |  |  | |
| *Sphaerocystis schroeteri* Chodat | CL63 |  |  | |  |  |  |  | |
| *Tetraedron caudatum* (Corda) Hansgirg | CL64 |  |  | |  |  |  |  | |
| *Tetraedron lobulatum* (Nägeli) Hansgirg | CL65 |  |  | |  |  |  |  | |
| *Tetraedron minimum* (A.Braun) Hansgirg | CL66 |  |  | |  |  |  |  | |
| *Tetrastrum* spp. | CL67 |  |  | |  |  |  |  | |
| *Treubaria triappendiculata* C.Bernard | CL68 |  |  | |  |  |  |  | |
| *Ulothrix aequalis* Kützing | CL69 |  |  | |  |  |  |  | |
| *Ulothrix cylindricum* Prescott | CL70 |  |  | |  |  |  |  | |
|  |  |  |  | |  |  |  |  | |
| CRYPTOPHYTA |  |  |  | |  |  |  | + | |
|  |  |  |  | |  |  |  |  | |
| *Cryptomonas curvata* Ehrenberg | CR1 |  |  | |  |  |  |  | |
| *Cryptomonas erosa* Ehrenberg | CR2 |  |  | |  |  |  |  | |
| *Cryptomonas marssonii* Skuja | CR3 |  |  | |  |  |  |  | |
| *Cryptomonas ovata* Ehrenberg | CR4 |  |  | |  |  |  | + | |
| *Cryptomonas* spp. | CR5 |  |  | |  |  |  | + | |
| *Katablepharis ovalis* Skuja | CR6 | - | +++ | |  | - |  |  | |
| *Komma caudate* (L.Geitler) D.R.A.Hill | CR7 |  |  | |  |  |  |  | |
| *Rhodomonas minuta* Skuja | CR8 |  |  | |  |  |  |  | |
|  |  |  |  | |  |  |  |  | |
| HETEROKONTOPHYTA (Diatoms) |  |  | + | |  |  |  |  | |
|  |  |  |  | |  |  |  |  | |
| *Amphipleura lindheimeri* Grunow | DI1 |  |  | |  |  |  |  | |
| *Cyclotella* large | DI2 |  |  | |  |  |  |  | |
| *Cyclotella* medium | DI3 |  |  | |  |  |  |  | |
| *Cyclotella* small | DI4 |  |  | |  |  |  |  | |
| *Fragilaria crotonensis* Kitton | DI5 |  |  | |  |  |  |  | |
| *Synedra acus* Kützing | DI6 |  |  | |  | + |  | + | |
| *Synedra delicatissima* W.Smith | DI7 |  |  | | ++ |  |  |  | |
| *Synedra ulna* (Nitzsch) Ehrenberg | DI8 | +++ |  | |  |  |  |  | |
|  |  |  |  | |  |  |  |  | |
| HETEROKONTOPHYTA (Chrysophytes) |  | + | |  |  | +++ |  | |  |
|  |  |  |  | |  |  |  |  | |
| *Chromulina* sp. 1 | CH1 |  |  | |  | +++ |  | +++ | |
| *Chromulina* sp. 2 | CH2 |  |  | |  |  |  | - | |
| *Chromulina* spp. | CH3 |  |  | |  |  |  |  | |
| *Chrysochromulina parva* Lackey | CH4 |  |  | |  | - |  |  | |
| *Mallomonas caudata* Iwanoff [Ivanov] | CH5 | ++++ |  | | +++ | +++ |  | +++ | |
| *Mallomonas urnaformis* Precott | CH6 |  |  | |  |  |  |  | |
| *Mallomonas* sp. 1 | CH7 |  | + | |  |  |  |  | |
| *Mallomonas* spp. | CH8 |  |  | |  |  |  |  | |
| *Ochromonas variabilis* Meyer | CH9 |  |  | |  |  |  |  | |
|  |  |  |  | |  |  |  |  | |
| EUGLENOPHYCEAE |  |  |  | |  | + |  |  | |
|  |  |  |  | |  |  |  |  | |
| *Euglena viridis* (O.F.Müller) Ehrenberg | EU1 |  |  | |  |  |  |  | |
| *Euglena* sp.1 | EU2 |  |  | |  |  |  |  | |
| *Phacus* sp.1 | EU3 |  |  | |  |  |  |  | |
| *Trachelomonas* sp.1 | EU4 |  |  | |  |  |  |  | |
|  |  |  |  | |  |  |  |  | |
